# IgM Flare in Anti-MAG Neuropathy Post Rituximab Treatment: A Clinical Case and a Systematic Review of the Literature

**DOI:** 10.3390/brainsci14121294

**Published:** 2024-12-22

**Authors:** Giovanni Siconolfi, Francesca Vitali, Maria Ausilia Sciarrone, Michelangelo Ardito, Valeria Guglielmino, Angela Romano, Giuseppe Granata, Gabriella Silvestri, Marco Luigetti

**Affiliations:** 1 Dipartimento Di Neuroscienze, Università Cattolica del Sacro Cuore, Sede Di Roma, 00168 Rome, Italy; siconolfig@yahoo.it (G.S.); vitali.francesca95@gmail.com (F.V.); a.sciarrone97@gmail.com (M.A.S.); michelangelo.ardito01@icatt.it (M.A.); guglielmino.valeria@gmail.com (V.G.); angela.romano12@gmail.com (A.R.); gabriella.silvestri@policlinicogemelli.it (G.S.); 2 Fondazione Policlinico Universitario A. Gemelli IRCCS, UOC Neurofisiopatologia, 00168 Rome, Italy; granata.gius@gmail.com; 3 Fondazione Policlinico Universitario A. Gemelli IRCCS, UOC Neurologia, 00168 Rome, Italy

**Keywords:** anti-MAG neuropathy, paraproteinemic neuropathy, rituximab, CD20, worsening, flare and Guillain–Barre

## Abstract

Background/Objectives: Anti-MAG polyneuropathy is a demyelinating peripheral neuropathy associated with IgM monoclonal gammopathies, particularly MGUS (monoclonal gammopathy of undetermined significance) and Waldenström macroglobulinemia. It is characterized by a subacute onset of distal sensory symptoms, with distal motor dysfunction typically appearing only in the later stages of the disease. The condition is caused by the presence of autoantibodies directed against myelin-associated glycoprotein, a structural protein of myelin. This leads to abnormalities in electrophysiological studies, such as markedly delayed distal latencies without conduction blocks or temporal dispersion of potentials. While rituximab (RTX) is the primary treatment, its efficacy is limited, with improvement seen in only 30–50% of patients. Recently, acute worsening of symptoms after RTX treatment has been increasingly reported. Methods: This systematic review compiles case reports and series from inception to June 2024 published on Scopus, PubMed or Cochrane, documenting acute exacerbations after RTX treatment in patients with anti-MAG polyneuropathy. Additionally, we present a case report from our institution that highlights this phenomenon. Results: We identified 13 clinical cases of acute deterioration in patients with anti-MAG polyneuropathy. Among these, eight patients (62%) achieved full recovery following additional treatment, while five patients (38%) did not return to their previous level of function. Plasmapheresis led to complete recovery in all four patients who received this intervention. Interestingly, many patients also experienced recovery after discontinuation of rituximab (RTX) treatment without the need for further therapeutic intervention. Conclusions: Acute clinical deterioration following RTX treatment in anti-MAG polyneuropathy is a possible occurrence. However, to date, no studies have assessed the true prevalence of this phenomenon. Further research is warranted to identify potential predictors of worsening following RTX treatment in this patient population.

## 1. Introduction

Anti-MAG neuropathy is a type of paraproteinemic neuropathy characterized by the presence of autoantibodies against myelin-associated glycoprotein (MAG), a structural myelin protein found in both the peripheral and central nervous systems [1]. Clinically, it typically manifests as a slowly progressive distal acquired demyelinating symmetric polyneuropathy (DADS), but atypical clinical presentations are also described [2,3,4]. Although the overall prognosis is generally favorable, some patients tend to develop significant levels of disability over time [5]. Historically, treatment primarily involved the use of chemotherapeutic agents. However, in recent years, anti-CD20 therapy, particularly Rituximab, has emerged as the first-line treatment to halt the progression of the disease [1]. Recently, alternative treatments for anti-MAG neuropathy have been proposed, particularly the use of tyrosine kinase inhibitors such as ibrutinib. These have shown promising results in a subgroup of patients with both anti-MAG neuropathy and Waldenström’s disease, specifically those with the MYD88 L265P mutation and wild-type CXCR4 [6,7]. In contrast, for patients who do not respond well to rituximab, the main rescue therapies are cytotoxic agents, including cyclophosphamide, fludarabine, and chlorambucil, despite these being associated with significant side effects [8,9]. Alternative treatments such as chronic IVIg and PLEX have shown conflicting evidence [10,11,12]. Many trials are currently underway, proposing various potential therapies, including novel tyrosine kinase inhibitors (such as albrutinib or zanubrutinib), lenalidomide, and glycopolymer therapy (poly(phenyl disodium 3-O-sulfo-β-d-glucopyranuronate)-(1→3)-β-d-galactopyranoside PPSGG), which involves selective binding and removal of autoantibodies [8]. Despite its widespread use, the efficacy of Rituximab is observed in only 30–50% of patients [13]. Moreover, several case reports have described acute worsening of symptoms in patients with anti-MAG neuropathy following Rituximab therapy, a phenomenon commonly referred to as flare IgM [14,15]. To further investigate this phenomenon, we conducted a systematic review of the literature. Additionally, we report a case of acute symptoms exacerbation in a patient with anti-MAG neuropathy treated with Rituximab, recently managed in our Department of Neurology.

## 2. Materials and Methods

This systematic review was performed and reported in accordance with the Cochrane Collaboration Handbook for Systematic Review of interventions and the Preferred Reporting Items for Systematic reviews and Meta Analysis (PRISMA) Statement guidelines. We systematically searched PubMed, Scopus and Cochrane from inception to June 2024 with the following search items: ‘anti MAG’, ‘anti MAG neuropathy’, ‘paraproteinemic neuropathy’, ‘rituximab’, ‘CD20’, ‘ worsening’, ‘ flare’, ‘AIDP’ and ‘Gullain Barre’. Two authors (G.S. and M.L.) independently extracted the data following predefined search criteria. We selected only case reports or case series of patients with a diagnosis of anti-MAG neuropathy who experienced an acute, rapid worsening of symptomatology after treatment with Rituximab. 

## 3. Results

**Figure 1:**
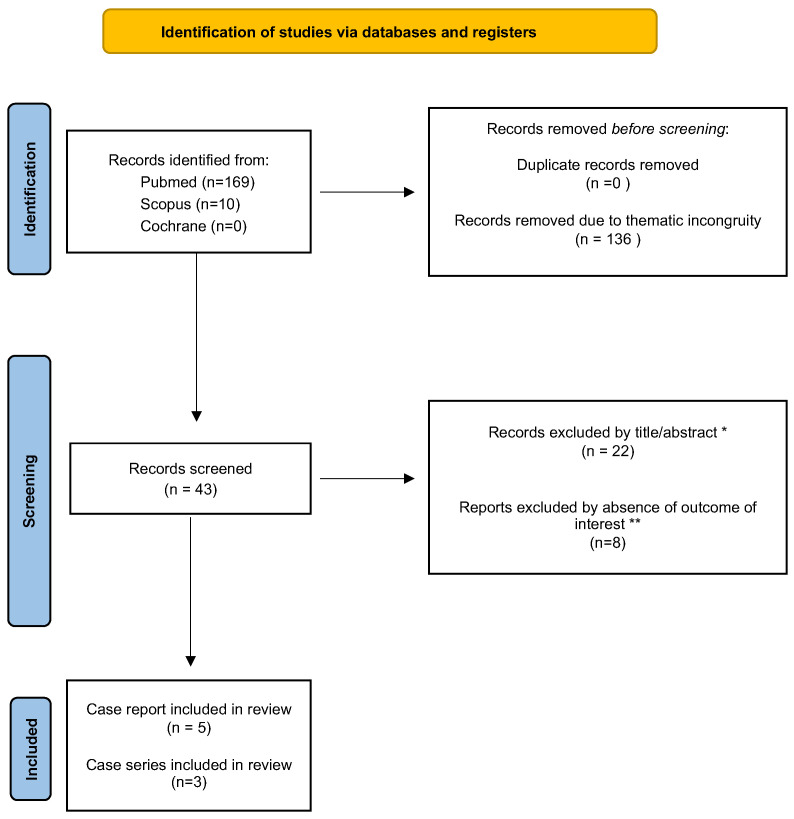
PRISMA chart of case series or case reports found in Pubmed, Scopus or Cochrane database with our search strategy. Legend: * indicate articles that did not meet the inclusion criteria (see Material and Methods); ** studies that did not include patients with anti-MAG neuropathy who worsened following Rituximab treatment.

The initial search yielded 179 results. After the removal of duplicate records and ineligible studies, 43 studies remained for full review. Of these, 8 studies were included, comprising 13 patients from 5 case reports and 3 case series (Figure 1). 

Most of the patients were male (7/13, 53%), with a mean age of 68 years. The mean duration of the disease was 2 years, and all 13 cases were diagnosed by an anti-MAG titer > 1000 BTU. Two out of 13 patients (15%) received Rituximab treatment following prior therapy with only IVIg, 1/13 (7%) after IVIg, steroids and cyclophosphamide, 1/13 (7%) after plasmapheresis, 1/13 (7%) after steroids, 1/13 (7%) after combination therapy of Rituximab with IVIg and 7/13 (53%) were treatment-naïve. Only 2 patients had a concomitant diagnosis of Waldenström’s macroglobulinemia, and all 13 patients had IgM monoclonal gammopathy. All patients were treated with the standard dosage of Rituximab (375 mg/m^2^) administered in weekly infusions. The mean number of Rituximab cycles before the worsening of symptoms was two, and the mean time to symptom deterioration was 19 days after the first infusion. Eight out of 13 patients (62%) experienced complete recovery after worsening, while 5/13 (38%) did not regain the same level of functional activity they had before the worsening of symptoms. Two out of 13 patients were treated with immunoglobulins alone, 1/13 with immunoglobulins and steroids, 3/13 with plasmapheresis, 1/13 with plasmapheresis and immunoglobulins, and 3/13 did not receive any treatment. For three patients, it was not specified whether any treatment was administered. Among the patients treated with plasmapheresis, all four achieved complete recovery (100%), while, in the group treated with only immunoglobulins or immunoglobulins and steroids, two out of three recovered fully. In only two patients, the anti-MAG and IgM titers increased after treatment, while, in the majority, these titers remained stable. In most cases, the worsening of symptoms primarily affected motor function (9/13, 69%), while sensory symptoms were involved in a smaller number of patients (7/13, 54%). Significant gait deterioration due to ataxia was also observed. In three patients, Rituximab was re-administered without any significant subsequent complications (Table 1).

## 4. Clinical Case

We present the case of a 61-year-old Caucasian female admitted to our Neurology department due to a rapid worsening of her neurological status. Her medical history included hypertension and Hashimoto’s thyroiditis. Additionally, 10 years before, she had undergone surgery for the removal of a benign breast lesion. In April 2024, she experienced a subacute onset of symmetrical hypoesthesia and paresthesia in her lower limbs, along with progressive difficulty in ambulation. To investigate these symptoms, an electroneurography was performed, which identified a sensory–motor demyelinating polyneuropathy with prolonged distal latency. During further investigations, a monoclonal IgM component was detected on electrophoresis and confirmed by immunofixation (kappa-restricted). Suspecting paraproteinemic neuropathy, anti-MAG autoantibodies were tested, and the patient was evaluated by a hematologist. The anti-MAG antibodies titer was 8351 BTU. A bone marrow biopsy was subsequently performed to further assess the monoclonal gammopathy, revealing an infiltration of lymphoplasmacytic cells (CD20+/CD79a+/CD23−/CD5−) and MYD88 L265P mutation, compatible with a diagnosis of lymphoplasmacytic lymphoma, indolent Waldenström macroglobulinemia. On 15 and 16 June, she initiated the first round of treatment (of a scheduled six cycles) with Rituximab and Bendamustine. However, on 25 June, she experienced an acute worsening of motor and sensory symptoms, particularly in her lower limbs, leading to a complete inability to ambulate independently. On 27 June, she was admitted to our hospital for further investigation. She was visited, and her first neurological examination showed a severe distal weakness in the lower limbs (MRC = 1 bilaterally on tibialis anterior muscle, MRC = 0 bilaterally on extensor hallucis longus). No motor deficit has been documented in the upper limbs. Additionally, hypoesthesia in the lower limbs with a stocking distribution, total areflexia in all four limbs, and hypopallesthesia were noted. The patient underwent a nerve conduction study (NCS), which revealed sensory–motor demyelinating polyneuropathy with a possible axonal component characterized by high distal latencies. Compared to the previous NCS, this new examination demonstrated a global reduction in nerve conduction velocity, decreased amplitude and morphology of desynchronized compound motor action potentials (Table 2).

An extensive blood laboratory workup, including serological tests for infection-related neuropathies, vitamin B12 and folate levels, HbA1c, and autoimmune screening, returned negative results. However, mild normochromic normocytic anemia was detected. The previously identified IgM monoclonal component with kappa restriction was confirmed through serum protein electrophoresis and serum and urine immunofixation. Specific tests for anti-gangliosides and cryoglobulins were also negative. Additionally, a TSH reflex test revealed a mild elevation with the presence of anti-thyroid peroxidase antibodies, consistent with a prior diagnosis of hypothyroidism. For neoplastic screening and to assess the extent of Waldenström’s disease, a total body CT scan was performed, revealing no signs of lymphoproliferative disease spread or direct infiltration. An MRI of the spine and brain was performed on 30 June. The brain MRI did not show any significant abnormalities, while the spine MRI revealed gadolinium enhancement in the cauda equina and conus medullaris regions, along with signal changes in the vertebral bodies, suggesting bone marrow infiltration. Later, on July 1st, the patient underwent a cerebrospinal fluid examination revealing severe hyperproteinorrachia, 248 mg/dL, (reference limit < 45 mg/dL) with five cells/mmc (reference limit < 5 cells/mmc), and no oligoclonal bands (Table 3). 

Given the neurological deterioration (total areflexia and acute motor deficit), gadolinium enhancement of the cauda equina and conus spine on MRI, and significant worsening of nerve conduction studies, our primary diagnostic suspicion was acute inflammatory demyelinating polyneuropathy (AIDP) or Guillain–Barré Syndrome (GBS). The patient was treated, starting on July 1st, with a 5-day course of IVIg (2 g/kg), resulting in an optimal clinical recovery. At discharge from rehabilitation on 10 September, two months after the onset of worsening, the neurological examination showed partial recovery of strength in the lower limbs (MRC score: Extensor Hallucis Longus 2 bilaterally, left Tibialis Anterior 3.5 and right tibialis anterior 4) without any relevant motor deficit in upper limbs and a reduction in paresthesia. Furthermore, ambulation was totally autonomous, although a mild degree of ataxia persisted. The nerve conduction study was repeated three months after admission, and it showed a mild improvement suggestive of a previous acute inflammation in resolution (Table 4). 

Considering the absence of clinical activity, the indolent course of Waldenström’s disease, and the recent worsening of symptoms following Rituximab treatment, it was decided, in agreement with the hematologist, to discontinue Rituximab therapy, proceeding only with clinical follow-up. The patient repeated the dosage of the anti-MAG antibodies three months after the acute onset of symptoms. The resulting titer was 80,000 BTU, ten times higher than the initial titer measured at the time of diagnosis.

## 5. Discussion

We present a case of an IgM flare in a patient with anti-MAG neuropathy following Rituximab treatment. Previous studies have shown that Rituximab is ineffective in 30–50% of patients with anti-MAG neuropathy. In our systematic review of the literature, we identified clinical cases of acute neurological worsening in patients with anti-MAG neuropathy after Rituximab therapy by conducting a comprehensive search in PubMed, Scopus and Cochrane. Acute neurological deterioration following Rituximab has also been reported in patients treated with the drug for other conditions [22,23,24]. We found 13 previously reported cases of acute neurological worsening after Rituximab therapy in patients with anti-MAG neuropathy. In all these cases, patients experienced significant neurological impairment with rapid, hyperacute progression. Typically, this worsening occurs after an average of two therapy cycles and tends to improve in the following weeks, even with the discontinuation of Rituximab alone. Among the cases we reviewed, plasmapheresis was the most effective treatment. Immunoglobulins and steroids were also beneficial in halting the progression of symptoms. However, this worsening is not associated with increased anti-MAG or IgM titers in the blood, as confirmed by previous studies. Unlike most reviewed cases, our patient showed a significant increase in anti-MAG antibody titers two months after the acute flare-up of symptoms. We did not measure antibody levels at the time of hospital admission, so it is possible that the anti-MAG titers fluctuated during this period. In some patients from the case series, retreatment with Rituximab after clinical improvement did not result in another acute flare of symptoms.

The underlying mechanism may be related to a pro-inflammatory response induced by Rituximab, involving the overproduction of certain cytokines, such as IL-6 [19,25,26]. Additionally, in vitro studies have suggested that a trogocytosis-like process induced by Rituximab could activate monocytes and macrophages through FcRII activation [27,28,29]. Further studies are needed to clarify which patient characteristics may predict a higher risk of IgM flares driven by Rituximab treatment. Moreover, considering the potential causative role of IL-6, it would be of interest to evaluate the possible use of anti-IL-6 antibodies in the acute management of IgM flare.

## 6. Conclusions

In conclusion, Rituximab is considered the first-line therapy for anti-MAG polyneuropathy, as it is for Waldenström’s macroglobulinemia and the associated neuropathy. However, growing evidence suggests it has limited effectiveness and carries a risk of acute worsening following administration. Further studies are needed to better understand the true risk of deterioration after Rituximab treatment in anti-MAG polyneuropathy and to determine whether specific patient profiles might indicate a higher risk.

## Figures and Tables

**Table 1:** Summary of data in patients with acute IgM flare after Rituximab treatment. Abbreviations: BTU: Buhlmann Titer Units; IVIg: Intravenous Immunoglobulins; F: Female; M: male; PE: Plasma Exchange; RTX: Rituximab; WM: Waldenström Macroglobulinemia; N: no; Y: yes.

Patient	Age and Sex	Disease Duration	WM	Pre-RTX Therapies	RTX Doses	Pre-Treatment Anti-MAG Levels	Post-Treatment Anti-MAG Levels	Pre-Treatment IgM (g/L)	Post-Treatment IgM (g/L)	Time of Deterioration	Symptoms	New Treatment	Recovery	RTX Retreatment
***1*** [14]	75, F	2 years	N	IVIg Steroids cyclophosphamide	4	1:400,000 (normal 1:12,800)	1:400,00	6.2	3.1	1 month	Ataxia, motor	/	Not complete	N
***2*** [15]	M	2 years	/	N	2	7180 BTU	5714 BTU	13	12	2 weeks	Motor, sensory	IVIg	Complete (5 days)	N
***3*** [15]	F	/	/	IVIg	2	49,786 BTU	25,713 BTU	3.9	3.2	2 weeks	Sensory, motor	RTX discontinuation	Not complete	N
***4*** [15]	M	5 years	/	N	3	1:409,600 (normal <1:1600)	1:102,400	4.7	4.6	3 weeks	Sensory	RTX discontinuation	Complete (3 weeks)	N
***5*** [16]	68, M	7 months	/	N	2	26,000 BTU	11,300 BTU	5.1	1.5	2 weeks	Ataxia, motor	PE	Complete (1 week)	Y
***6*** [16]	61, M	6 months	/	PE	1	38,943 BTU	36,420 BTU	1.7	1.1	9 days	Ataxia, sensory, motor	PE	Complete	Y
***7*** [17]	85, M	2 years	/	N	2	12,800 BTU	51,200 BTU	1.9	2.7	2 weeks	Ataxia, motor	RTX discontinuation	Complete (6 weeks)	N
***8*** [18]	53, F	1 year	/	N	2	1:102,400	/	4.4	3.9	2 weeks	/	IVIg	Complete	N
***9*** [19]	64, F	8 years	Y	N	4	144,000 BTU	174,000 BTU	5	7.9	3 months	Ataxia, motor, sensory	RTX discontinuation	Not complete	N
***10*** [20]	69, M	1 year	N	IVIg	1	86,567 BTU	68,234 BTU	5.2	4.3	2 days	Ataxia, motor, sensory	IVIg + steroids	Not complete (8 months)	N
***11*** [20]	62, F	10 months	N	N	1	60,000 BTU	36,452 BTU	3.3	2.8	1 weeks	Ataxia, sensory	/	Complete (1 month)	Y
***12*** [20]	65, M	7 years	N	RTX, IVIg, steroids	3	1366 BTU	3478 BTU	/	/	1 weeks	Ataxia, motor	PE	Not complete	N
***13*** [21]	72, M	5 months	Y	Steroids	1	/	/	1.1	1.5	1 weeks	Ataxia, sensory, motor	PE + IVIg	Complete (1 month)	N
***Our case***	61, F	5 months	Y	N	1	8351 BTU	80,000 BTU	/	/	2 weeks	Ataxia, sensory, motor	IVIg	Complete (2 months)	N

**Table 2:** Nerve conduction studies at the time of neurological deterioration.

*Nerve*	*Tract*	*Morphology*	*MCV* (m/s)	*dL* (ms)	*CMAP* (mV)
R Median	E-W	Desynchronized	36 (≥45)		3.3 (≥5)
	W-APB			4.8 (≤4)	6.7 (≥5)
R Ulnar	Ax-Ab. E	Desynchronized	50 (≥45)		2.3 (≥5)
	Ab. E-Bel. E		33 (≥45)		2.4 (≥5)
	E-W		33 (≥45)		2.5 (≥5)
	W-ADM			4.4 (≤3)	3.7 (≥5)
R Deep Peroneal	FH-Ankle	Desynchronized	12 (≥40)		0.1 (≥2)
	Ankle-EDB			11.1 (≤4)	0.2 (≥2)
R Tibialis	LM-AH	Desynchronized		12.8 (≤5)	0.2 (≥5)
*Nerve*			*SCV* (m/s)		*SNAP* (μV)
R Sural	A-SURA				absent
L Sural	A-SURA				absent
R Radial	IF-W	Desynchronized	36 (≥45)		1.2 (≥5)
R Median	IF-W	Desynchronized	36 (≥45)		0.8 (≥5)
R Ulnar	VF-W	Desynchronized	29 (≥50)		0.2 (≥5)

Legend to the table: MCV, motor conduction velocity; dL, distal latency; CMAP, compound muscle action potential; SCV, sensory conduction velocity; SNAP, sensory nerve action potential; R, right; L, left; Ax, axilla; E, elbow; W, wrist; APB, abductor pollicis brevis; Ab. E-Bel. E, above elbow-below elbow; ADM, abductor digiti minimi; A, ankle; FH, fibula head; EDB, extensor digitorum brevis; LM, lateral malleolus; AH, abductor hallucis; IF, first finger; VF, fifth finger. Values in brackets indicate reference values.

**Table 3:** Excursus of the various exams performed during admission to the department.

Exams	Main Results
Blood laboratory workup Biochemistry exams Serological tests for infection-related neuropathies (Borrelia Burgdorferi, Toxoplasma, CMV, Echovirus, HSV1/2, Measles, Mumps, VZV, EBV) Autoimmune screening (ANA, ENA, SS-A, SS-B, dsDNA, Sm, RNP, pANCA, cANCA, LKM, SCL-70) Protein Electrophoresis Serum and urine immunofixation IgM and IgG anti gangliosides and Cyoglobulins	Mild normochromic normocytic anemia Past infections or vaccinations (positive IgG Toxoplasma gondii, IgG CMV, IgG measles, IgG HSV, IgG Mumps, IgG VZV). Absence of active infections (none positive for IgM) Autoimmune screening resulted negative Monoclonal gammopathy Monoclonal IgM, kappa restricted Negative
Lumbar puncture Cerebrospinal fluid examination Serological and molecular microbiological exams (Borrelia Burgdoferi, CMV, Echovirus, HSV1/2, Measles, Mumps, VZV, Mycobacterium tuberculosis) Cytological exam	Severe hyperproteinorrachia, 248 mg/dL, (reference limit < 45 mg/dL) with 5 cells/mmc (reference limit < 5 cells/mmc), and no oligoclonal bands. IgG in cerebrospinal fluid 186 mg/L (reference limit < 40 mg/L), Albumin in cerebrospinal fluid 1740 mg/L (reference limit < 200), Albumin liquor/serum ratio 43.8 (reference limit < 5.5). Previous data are suggestive of blood–brain barrier damage Serological and molecular exams resulted negative Not atypical lymphocytes
Radiological exams Total body CT scan Spine MRI with gadolinium	No signs of lymphoproliferative disease spread (not an increase in lymphadenopathies, organomegaly) or direct infiltration Gadolinium enhancement in the cauda equina and conus medullaris regions, along with signal changes in the vertebral bodies, suggesting bone marrow infiltration

Legend to the table: CMV, cytomegalovirus; HSV, herpes simplex virus; VZV, varicella–zoster virus; EBV, Epstein–Barr virus; ANA, antinuclear antibodies; ENA, extractable nuclear antigens; SS-A, anti-Sjögren’s syndrome-related antigen A autoantibodies; SS-B, anti-Sjögren’s syndrome-related antigen B autoantibodies; dsDNA, double-strand DNA; Sm, Smith autoantibodies; RNP, ribonucleoprotein antibodies; pANCA, perinuclear anti-neutrophil cytoplasmic antibodies; c-ANCA, cytoplasmic anti-neutrophil cytoplasmic antibodies; LKM, anti-liver–kidney microsomal antibody; SCL-70, anti-topoisomerase I.

**Table 4:** Follow-up nerve conduction studies.

*Nerve*	*Tract*	*Morphology*	*MCV* (m/s)	*dL* (ms)	*CMAP* (mV)
R Median	E-W	Desynchronized	38 (≥45)		6.3 (≥5)
	W-APB			6.1 (≤4)	6.4 (≥5)
R Ulnar	Ax-Ab. E	Desynchronized	52 (≥45)		4.1 (≥5)
	Ab. E-Bel. E		28 (≥45)		4.5 (≥5)
	E-W		33 (≥45)		4.3 (≥5)
	W-ADM			4 (≤3)	5.7 (≥5)
R Deep Peroneal	FH-Ankle	Desynchronized	12 (≥40)		0.1 (≥2)
	Ankle-EDB			9.2 (≤4)	0.2 (≥2)
R Tibialis	LM-AH	Desynchronized		9.7 (≤5)	0.2 (≥5)
*Nerve*			*SCV* (m/s)		*SNAP* (μV)
R Sural	A-SURA	Desynchronized	37 (≥40)		3.3 (≥8)
R Radial	IF-W	Desynchronized	34 (≥45)		1.5 (≥5)
R Median	IF-W	Desynchronized	30 (≥45)		1.3 (≥5)
R Ulnar	VF-W	Desynchronized	38 (≥50)		0.6 (≥5)

Legend to the table: MCV, motor conduction velocity; dL, distal latency; CMAP, compound muscle action potential; SCV, sensory conduction velocity; SNAP, sensory nerve action potential; R, right; L, left; Ax, axilla; E, elbow; W, wrist; APB, abductor pollicis brevis; Ab. E-Bel. E, above elbow-below elbow; ADM, abductor digiti minimi; A, ankle; FH, fibula head; EDB, extensor digitorum brevis; LM, lateral malleolus; AH, abductor hallucis; IF, first finger; VF, fifth finger. Values in brackets indicate reference values.

## Data Availability

These data were derived from the following resources available in the public domain: PubMed, Cochrane and Scopus.
